# Comparison of Long-Term Outcomes in Early-Stage Endometrial Cancer: Robotic Single-Site vs. Multiport Laparoscopic Surgery

**DOI:** 10.3390/jpm14060601

**Published:** 2024-06-04

**Authors:** Heeju Kang, Hyewon Chung, Seungmee Lee, Tae-Kyu Jang, So-Jin Shin, Sang-Hoon Kwon, Chi-Heum Cho

**Affiliations:** Department of Obstetrics and Gynecology, Keimyung University School of Medicine, Daegu 42601, Republic of Korea; rose6994@naver.com (H.K.); hyewonny81@naver.com (H.C.); seungmeemd@gmail.com (S.L.); tiber0103@dsmc.or.kr (T.-K.J.); hope2014@dsmc.or.kr (S.-J.S.); ksh1999@dsmc.or.kr (S.-H.K.)

**Keywords:** endometrial cancer, staging operation, single site, robotic surgery

## Abstract

The purpose of this study was to establish the noninferiority of robotic single-site (RSS) surgery compared with multiport laparoscopic (MPL) surgery in surgical outcomes and overall survival for early endometrial cancer. This study was conducted retrospectively in a single center and included 421 patients who underwent either RSS (*n* = 146) or MPL (*n* = 275) surgery between 2014 and 2022. In terms of perioperative outcomes, the RSS group had a longer operating time than the MPL surgery group (mean (standard deviation [SD]) RSS 97.55 [29.79] vs. MPL 85.56 [26.13], *p* < 0.001). However, no significant differences in estimated blood loss or perioperative complications were found between the groups (*p* = 0.196 and *p* = 0.080, respectively). The patients in the RSS group were discharged earlier than those in the MPL group (mean [SD]): 4.06 [3.24] vs. 9.39 [4.76], *p* < 0.001). Regarding oncologic outcomes, no significant differences in the type of therapy, disease stage, tumor grade, histopathological type, or lymphovascular invasion were found between the groups. No statistically significant differences were found in the disease-free (*p* = 0.27) and overall survival rates (*p* = 0.5) either. In conclusion, this study suggests that RSS and MPL surgery are both safe and effective options for staging operations in patients with early-stage endometrial cancer.

## 1. Introduction

Endometrial cancer is the sixth most common cancer among women, with 41,700 newly diagnosed cases worldwide in 2020 [1]. Its estimated age-standardized incidence rate is 8.3 per 100,000 women. In endometrial cancer, surgery is the first diagnostic step, as the biopsy result defines the final diagnostic stage. Surgical staging of endometrial cancer includes total hysterectomy and bilateral salpingo-oophorectomy with lymph-node assessment [2].

Minimally invasive surgery (MIS) is performed globally, and the LAP2 trial showed the safety and feasibility of laparoscopic surgery in staging operations for endometrial cancer [3]. Compared with traditional laparotomy, laparoscopic surgery is preferable owing to its shorter operation time, shorter hospital stay, and less intraoperative blood loss; thus, it is now considered the standard treatment for endometrial cancer [4,5,6].

After 2000, a robotic approach was introduced as an MIS option. As the number of multiport robotic surgery cases has increased, many attempts to perform a single-site approach have been made [7,8]. In robotic surgery, a single-site system is more applicable than laparoscopy in terms of articulation. Attempts have been made to apply a single-site robotic approach in staging operations for endometrial cancer, and recently, cases using the approach have been increasing [9].

In gynecological cases, being a female patient may mean having other influencing factors in the choice of surgical procedure, such as cosmetic issues. Female patients are more concerned about surgical scars than surgery itself. In recent years, as patients have resisted large abdominal incisions for cancer surgery, cosmetic factors such as smaller and fewer incisions have tended to become crucial for choosing a surgical method. Owing to its cosmetic benefits, less pain, and faster recovery, single-site surgery is increasingly preferred, and many clinical studies have published comparisons between the outcomes of single-port and multiport surgeries.

Robotic single-site (RSS) surgery is the most satisfactory surgical method in terms of patient aesthetics and recovery. Assuming that the outcomes of RSS surgery are proven to be not inferior to multiport laparoscopic (MPL) surgery, the future trend of surgery will lean toward a robotic platform, especially the single-site method.

Studies have demonstrated that RSS surgery is feasible, safe, and associated with fast patient recovery, but long-term data on its oncological outcomes are lacking [10]. In our institution, we began applying single-site surgery using a DaVinci Si^®^ starting in 2014, and more cases have been addressed using a DaVinci Xi^®^ and a DaVinci SP (single-port platforms). With these cumulated cases, we compared perioperative outcomes and oncologic outcomes of the RSS and MPL surgeries performed at our center.

## 2. Materials and Methods

In this retrospective study, we enrolled all patients who had been diagnosed or histologically confirmed as having early-stage endometrial cancer (stages IA and IB) and had undergone a staging operation using either the conventional laparoscopic method or the single-site robotic method in the Department of Obstetrics and Gynecology at Keimyung University Dongsan Medical Center, Republic of Korea, between March 2014 and December 2022. This study was conducted in accordance with the Declaration of Helsinki and approved by the Institutional Review Board of Keimyung University (IRB FILE No.: 2023-03-006). All the patients had been informed about the RSS techniques and the MPL surgery and their benefits, as well as the related risks of possible laparoscopic or laparotomic conversion. All data were collected from the patients’ electronic medical records.

The patients’ demographic parameters were age at diagnosis, body mass index (BMI), concurrent cancer, hypertension or diabetes, previous operations, and menopause status. Operation time, estimated blood loss, postoperative hospital stay, and perioperative complications were recorded. The clinical and pathological variables considered were the type of therapy, operative method, cancer stage (2018 International Federation of Gynecology and Obstetrics [FIGO] stages), tumor grade, histopathological type, lymphovascular invasion, and number of pelvic lymph nodes obtained. Overall survival (OS) and disease-free survival (DFS) were assessed using these data. DFS was defined as the period from the first diagnosis to the recurrence of cancer, death, or failure to follow up. Kaplan–Meier curves were plotted to assess OS and DFS.

The Shapiro–Wilk test was used to test the normality of the data. The patients’ characteristics were described as absolute frequencies with percentages for the nominal variables and as means (standard deviation [SD]) for the continuous variables. The RSS and MPL groups were compared using the Mann–Whitney or Student t test for the continuous variables, and the chi-square or Fisher exact test for the categorical variables.

Univariate and multivariate Cox proportional hazards models were also used to assess the effects of surgery type (laparoscopic vs. robotic), age (≤65 vs. >65), BMI (>30 vs. ≤30), cancer stage (stages 1 and 2 vs. stages 3 and 4), tumor grade (grade 1 vs. grades 2 and 3), histopathology (endometrioid vs. non-endometrioid), lymphovascular invasion (negative vs. positive), and postoperative treatment (follow-up vs. radiotherapy vs. chemotherapy vs. concurrent chemoradiotherapy) on OS and DFS. A *p* value ≤ 0.05 was considered statistically significant. The statistical analysis was performed using SPSS software (Version 25.0; SPSS Inc., Chicago, IL, USA) and R software (Version 4.2.1).

## 3. Results

A total of 421 patients with impressions of stage IA and IB endometrial cancer who had undergone MIS were selected. Of these patients, 146 underwent RSS surgery and 275 underwent MPL surgery. Table 1 shows the characteristics of the patients in each group. Significant differences between the RSS and MPL groups were found in terms of age (51.95 [8.28] vs. 55.13 [11.27], *p* = 0.001) and BMI (24.82 [4.84] vs. 26.57 [5.43], *p* = 0.001). No significant difference in history of concurrent cancer was found (*p* = 0.0.279). However, the two groups significantly differed in histories of diabetes (13 [8.9%] vs. 45 [16.4%], *p* = 0.035), hypertension (30 [20.5%] vs. 95 [34.5%], *p* = 0.003), and previous abdominal operation (66 [45.2%] vs. 88 [32.0%], *p* = 0.007).

Postoperative treatment and the extent of lymph node dissection showed no significant differences between the groups. However, oophorectomy status was significantly different between the two groups (Table 2). When pathological outcomes were analyzed and compared between the two groups, grade and histopathology showed no difference (*p* = 0.135 and *p* = 0.296, respectively). However, the final FIGO stage and lymphovascular space invasion were significantly different, presenting the MPL group as more severe than the RSS group. The number of pelvic lymph nodes was significantly higher in the MPL group than in the RSS group (mean [SD]: 9.58 [5.22] vs. 13.79 [7.57], *p* < 0.001).

**Figure 1:**
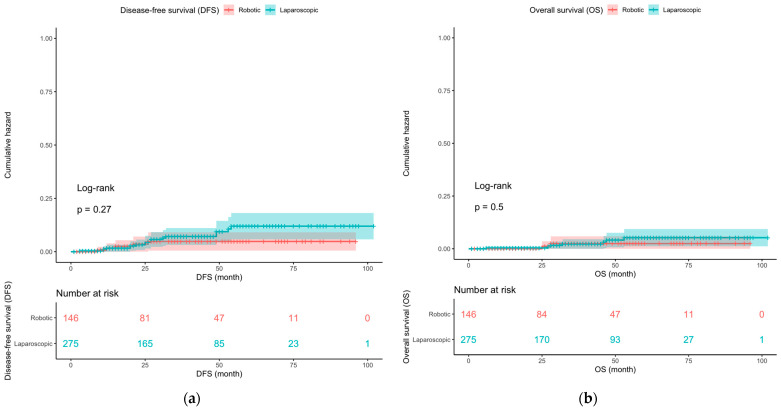
(**a**) Disease-free survival (DFS) of the patients in the robotic single-site (RSS) surgery group vs. the multiport laparoscopic (MPL) surgery group, *p* value = 0.27; (**b**) overall survival (OS) of the patients in the robotic single-site (RSS) surgery group vs. the multiport laparoscopic (MPL) surgery group, *p* value = 0.5.

The total intraoperative time differed significantly between the RSS and MPL groups (mean [SD]: 97.55 [29.79] vs. 85.56 [26.13], *p* < 0.001; Table 3). We found no significant differences in intraoperative estimated blood loss (*p* = 0.303) or total postoperative complications (*p* = 0.056). However, we detected more perioperative complications such as vaginal cuff disruption, rectum tear, and incisional hernia, each comprising one case, in the RSS group. The patients in the RSS group were discharged earlier than those in the MPL group (mean [SD]: 4.06 days [3.24] vs. 9.3 days [4.7], *p* < 0.001) (Figure 1).

The Kaplan–Meier survival analysis revealed no statistically significant differences in DFS (*p* = 0.27) or OS (*p* = 0.5) between the two groups.

Univariate and multivariate analyses were performed on the overall study population (Table 4). In terms of DFS, the univariate and multivariable analyses showed no significant differences between the surgical approaches (adjusted hazard ratio [aHR] = 0.57; 95% confidence interval [CI], 0.21–1.56; *p* = 0.275 vs. aHR = 1.19; 95% CI, 0.39–3.65; *p* = 0.765). In the univariate analysis, the influencing factors were age (aHR = 2.48; 95% CI, 1.01–6.08), tumor grade 3 (aHR = 3.62; 95% CI, 1.1–11.9), non-endometrioid histopathology (aHR = 4.63; 95% CI, 1.94–11.07), and lymphovascular invasion (aHR = 3.02; 95% CI, 1.29–7.06). However, the multivariate analysis revealed statistically significant differences only in positive lymphovascular space invasion (aHR = 5.86; 95% CI, 1.79–19.17).

OS showed no significant differences between the surgical approaches in the univariate analysis (aHR = 0.501; 95% CI, 0.12–2.81; *p* = 0.501) or the multivariate analysis (aHR = 1.35; 95% CI, 0.22–8.5; *p* = 0.746). Stage III (aHR = 7.32; 95% CI, 1.41–37.93), tumor grade 3 (aHR = 6.57; 95% CI, 1.1–39.37), histopathology (aHR = 6.22; 95% CI, 1.67–23.25), lymphovascular space invasion (aHR = 2.38; 95% CI, 0.57–9.95), and adjuvant radiotherapy (aHR = 10.15; 95% CI, 2.21–6.74) were identified as effective factors in the univariate analysis, but no significant factor was found in the multivariate analysis.

## 4. Discussion

In the treatment of endometrial cancer, staging operations, including total hysterectomy, bilateral salpingo-oophorectomy, and lymph node dissection, are the first step in simultaneous diagnosis and treatment. Various surgical methods can be used to treat endometrial cancer, but ultimately, there should be no difference in overall survival.

As mentioned in the introduction, the noninferiority of MIS for endometrial cancer has been consistently demonstrated in various studies comparing laparotomy to MIS. Among these studies, the LAP2 trial was the most representative and the largest randomized controlled trial study, with 2616 subjects included [3]. The patient group in the LAP2 study was similar to the patient group in this study (patients with clinically diagnosed stage I and II endometrial cancer). The 5-year OS of the patients who underwent MIS was 89.8%, proving the feasibility, safety, and oncological outcomes of MIS. MIS has an advantage over laparotomy in terms of perioperative outcomes. Furthermore, in recent studies, the feasibility of MIS has been demonstrated for advanced cancer stages such as FIGO stage IIIc [11,12].

Robotic surgery, which was introduced in the early 2000s after FDA approval, built upon the laparoscopic approach by enhancing surgical precision and reducing physical strain on surgeons by improving three-dimensional visibility and range of motion using articulated instruments. Therefore, many attempts have been made to apply the robotic approach in benign and malignant gynecological surgeries, with the number of cases increasing in recent times. By 2021, 6730 Da Vinci Surgical System units had been introduced in 69 countries. Between 2012 and 2022, the proportion of surgical procedures performed in the United Sates using robotic technology surged from 0% to 22%. It is anticipated that the proportion of robotic surgical procedures will continue to increase in the future, enhancing its accessibility. According to our review of the literature, two randomized trials have compared Robotic MIS with total abdominal hysterectomy (TAH) and conventional laparoscopic MIS (LMIS) in patients with early-stage endometrial cancer.

A population-based prospective cohort study by Jørgensen et al. that was published in March 2019 evaluated survival outcomes after a nationwide introduction of robotic surgery for women with early-stage endometrial cancer (FIGO stage I–II) from January 2005 to June 2015 in Denmark [13]. According to this study, robotic surgery for early-stage endometrial cancer was associated with improved patient survival regardless of age, BMI, America Society of Anesthesiologists score, comorbidity, smoking, socioeconomic status or histopathological risk. These findings elucidated the impact of robotic surgery on both survival rates and perioperative outcomes in women diagnosed with early-stage endometrial cancer within a nationwide context. Their study also highlighted the importance of considering both the clinical effectiveness and cost-effectiveness of robotic surgery in the management of early-stage endometrial cancer at the population level.

Another retrospective cohort study by Corrado et al., which was conducted for the National Cancer Institute of Rome, compared three groups of patients who underwent laparotomic, laparoscopic, and robotic surgeries for endometrial cancer. This study also demonstrated that MIS had better surgical outcomes than open surgery, and that robotic surgery was superior to laparoscopic surgery in terms of intraoperative and postoperative complications, conversion rate, hospitalization period, and reoperation. The recurrence and survival outcomes were similar between the three groups [14]. Studies have generally shown robotic surgery to be superior or at least equivalent to traditional methods in terms of surgical outcomes, complication rates, and length of hospital stays.

However, these studies were conducted using multiport, not single-site, robots. Most of these studies reported limited results for long-term oncological outcomes because their follow-up periods were too short to investigate survival. RSS surgery has been available, and the use of single-site surgery, which has many advantages over multiple robotic arms surgery, has been gradually increasing for the treatment of endometrial cancer. In this study, we analyzed the survival rate and perioperative risks of RSS surgeries performed since 2014 on patients with endometrial cancer.

We first implemented a robotic staging operation using a Da Vinci Si^®^ (Intuitive Surgical, Sunnyvale, CA, USA) in 2014. We published our initial experiences from 2014 to 2015 with robotic single-site surgery for patients with early-stage endometrial cancer and demonstrated its feasibility and safety [15]. In our center, the number of cases has steadily increased since the introduction of the Da Vinci Xi^®^ in 2019 and the Da Vinci SP^®^ in 2022.

Over the years, we have collected enough cases of RSS staging operations for endometrial cancer and the traditional MPL method, which was also continuously performed during the same period, to enable comparison. In this study, we compared the surgical and oncological outcomes of RSS and MPL surgeries in patients who underwent staging operations for endometrial cancer diagnosed as stage IA and IB.

The results of this study show that the RSS staging operation is feasible and safe compared with the MPL approach. First, in terms of postoperative outcomes, the RSS surgery showed a significantly shorter hospitalization period, and no significant differences were found between the two groups in estimated blood loss or postoperative complications. The shorter period to hospital discharge in the RSS group may be due to the effect of less wound site pain with less tissue injury resulting from the difference in the number of trocar sites.

The feature of single-site surgery that distinguishes it from multiport surgery is the number of surgical incisions it requires. According to Fagotti et al. [16], patients’ perception of surgical scars is not simply a “cosmetic problem”, but rather reflects a body image that brings to mind memories and experiences of cancer. Owing to this minimally invasive feature, single-site surgery, which leaves a scar only in the umbilicus, could be a great alternative solution for patients. The number of incisions addresses not only cosmetic concerns but also invasiveness, which is also related to the rapid recovery of the patients who undergo robotic surgery.

However, the intraoperative time was statistically significantly longer in the RSS surgery group. This time difference between the two groups can be interpreted based upon the specifications of the robotic model used. When RSS surgery was subdivided into the Da Vinci Si^®^, Xi^®^, and SP^®^ models and analyzed, the intraoperative time was shorter or similar to that of the MPL surgery group except with the Da Vinci Si^®^, the earliest model. Considering this, we can predict that as the robotic platform is upgraded, the robotic method will show a similar postoperative outcome that is not inferior to that of MPL.

In terms of oncological outcomes, the DFS and OS were compared between the MPL and RSS groups. In our Kaplan–Meir analysis, recurrence and mortality rates were higher in the robotic approach but did not show statistical significance.

This study has limitations owing to its retrospective nature. Better results could be obtained by designing a prospective study to eliminate statistical differences between the groups in the future. In addition, in this study, as shown in Table 1, significant differences in age, BMI, and underlying disease were found in the characteristics of the two patient groups. In our study design, we compared patients who underwent robotic and laparoscopic surgeries without considering their characteristics. This was based on the assumption that these factors would not significantly impact patient prognosis, especially given the relatively low prevalence of severe obesity and its related complications in South Korea. In addition, univariate and multivariate analyses of DFS and OS were performed for each covariate, but no statistical significance was found (Table 4). Nevertheless, for further advanced research, it might be beneficial to either select similar characteristics for both groups or consider subgrouping.

This study acknowledges that one limitation of robotic surgery is its high cost, which makes patients and surgeons hesitate to choose it. Although robotic surgery is a high-cost option compared with laparoscopy, the ultimate goal of this study was to compare single-site surgery with multiport surgery. As shown in a previous study conducted in our center, the total hospital charge, including hospitalization, surgical equipment, and costs for a single-site approach is more cost-effective than that for a multiport approach [17]. Moreover, in South Korea, where medical insurance coverage is extensive and medical expenses are relatively low, patients can easily access the robotic option. Thus, the number of robotic systems has increased geographically. As of 2019, 58 hospitals in Korea, with a total of 85 robotic systems, including our institution’s three major robotic systems, have been actively using robotic surgery. Many individuals can now consider robotic surgery as a reasonable and accessible option, given the establishment of numerous robotics training centers.

It is also a widely known fact that the robotic approach has a steep learning curve and has to be performed by trained medical staff. Considering the nearly decade-long history of robotic surgical procedures, it is reasonable to assume that most robotic surgeons have gained proficiency in performing stable surgeries. Currently, RSS surgery is infrequently applied to patients with early-stage endometrial cancer. In most centers, including our hospital, laparoscopy or laparotomy is mainly performed for patients with more advanced cancer stages. Robotic surgery has limitations, and several complementary points must be addressed to treat advanced cancer stages. This study also targeted patients with early-stage (stages IA and IB) cancer, accounting for most cases (RSS, 81.5% and MPL, 86.5%). Some patients in the RSS group were diagnosed postoperatively with stage III disease, which was higher than the preoperative diagnosis. When enough cases are accumulated, a follow-up and comparative study on their outcomes will also be meaningful.

Compared with previous research, this study had a distinctive advantage in that it had a comparably longer follow-up period, with many cases using the RSS method for endometrial cancer, and it compared DFS and OS, unlike previous research that compared laparoscopic and robotic methods. The superiority of the perioperative outcomes, noninferiority of the 5-year survival rate, and DFS time of the patients who underwent the RSS approach were demonstrated in this study. This study provides evidence for recommending the RSS approach as an option for patients with clinically diagnosed early-stage endometrial cancer.

In summary, the incorporation of robotic surgery into the treatment protocol for endometrial cancer reflects a significant advancement in surgical technology, offering not only improved safety profiles, reduced length of hospital stay, and potentially better overall patient outcomes, but also a long-term OS rate. However, ongoing research, including prospective studies and randomized controlled trials, is necessary to continue evaluating its efficacy and optimizing its use.

## 5. Conclusions

Through various studies, MIS for endometrial cancer has already been accepted as superior to laparotomy in terms of short-term perioperative outcomes, and comparable in terms of risk of recurrence. As shown in this study, RSS surgery maximizes the perioperative benefit of MIS compared with MPL, while its effectiveness is proven for oncological outcomes. This result can be part of the evidence to provide patients with when introducing RSS as an option for treating early endometrial cancer.

## Figures and Tables

**Table 1:** Patient characteristics (total 421).

	Robotic (*n* = 146)	Laparoscopic (*n* = 275)	*p*-Value
Age (years), mean ± standard variation	51.95 (±8.28)	55.13 (±11.27)	0.001
BMI (kg/m^2^), mean ± standard variation	24.82 (±4.84)	26.57 (±5.43)	0.001
Concurrent cancer Breast cancer Cervix cancer Gastrointestinal tract cancer Ovary cancer Other	2 (1.4%) 3 (2.1%) 1 (0.7%) 0 (0%) 1 (0.7%)	7 (2.5%) 1 (0.4%) 4 (1.5%) 2 (0.7%) 8 (3.0%)	0.279
Diabetes (%)	13 (8.9%)	45 (16.4%)	0.035
Hypertension (%)	30 (20.5%)	95 (34.5%)	0.003
Previous abdominal operation (%)	66 (45.2%)	88 (32.0%)	0.007
Menopause at diagnosis	71 (48.6%)	163 (59.3%)	0.036

**Table 2:** Pathologic findings and adjuvant treatments.

	Robotic (*n* = 146)	Laparoscopic (*n* = 275)	*p*-Value
Adjuvant treatment Follow up RTx CCRTx * CTx *	105 (71.9%) 6 (4.1%) 25 (17.1%) 10 (6.8%)	176 (64.0%) 22 (8.0%) 49 (17.8%) 28 (10.2%)	0.234
Operative Method			
Oophrectomy None Unilateral Bilateral	13 (8.9%) 38 (26.0%) 95 (65.1%)	45 (16.4%) 33 (12.0%) 196 (71.5%)	<0.001
Lymph node dissection None Sentinel lymph node dissection BPND * BPND * + PAND *	14 (9.6%) 0 (0.0%) 113 (77.4%) 19 (13.0%)	11 (4.0%) 4 (1.5%) 224 (81.5%) 36 (13.1%)	0.062
Biopsy Result			
FIGO staging Stage IA Stage IB Stage II Stage III No data	108 (74.0%) 11 (7.5%) 12 (8.2%) 11 (7.5%) 4 (2.7%)	187 (68.0%) 51 (18.5%) 13 (4.7%) 19 (6.9%) 5 (1.8%)	0.030
Grade Grade 1 Grade 2 Grade 3 other	85 (58.2%) 37 (25.3%) 16 (11.0%) 8 (5.5%)	129 (46.9%) 80 (29.1%) 41 (14.9%) 25 (9.1%)	0.135
Histopathology Endometrioid Mucinous Serous Clear cell ESS * Mixed Others	134 (91.8%) 0 (0.0%) 3 (2.1%) 2 (1.4%) 2 (1.4%) 4 (2.7%) 1 (0.7%)	233 (84.7%) 1 (0.4%) 13 (4.7%) 8 (2.9%) 4 (1.5%) 16 (5.9%) 0 (0.0%)	0.296
Lymphovascular invasion No Yes Undetermined	117 (80.1%) 23 (15.8%) 6 (4.1%)	208 (75.6%) 64 (23.3%) 3 (1.1%)	0.031
Pelvic lymph node	9.58 (±5.22)	13.79 (±7.57)	<0.001

* RTx, radiotherapy; CCRT, chemoradiotherapy; CTx, chemotherapy; BPND, bilateral pelvic lymph node dissection; PAND, para-aortic lymph node dissection; ESS, endometrial stromal sarcoma.

**Table 3:** Postoperative outcome.

	Robotic Method (*n* = 146)	Laparoscopic Method (*n* = 275)	*p*-Value
Operative time (min)	97.55 (±29.79)	85.56 (±26.13)	<0.001
Perioperative complications Incisional hernia Lymphocele Rectum tear Vaginal cuff bleeding Vaginal cuff disruption Vaginal cuff infection	1 (0.7%) 1 (0.7%) 1 (0.7%) 1 (0.7%) 3 (2.1%) 0	0 1 (0.4%) 0 1 (0.4%) 0 1 (0.4%)	0.080
Postoperative hospital stay (day)	4.06 (±3.24)	9.39 (±4.76)	<0.001
Estimated blood loss (mL)	134.11 (±39.03)	127.58 (±63.80)	0.196

**Table 4:** Univariate and multivariate analysis of patients’ characteristics according to disease-free survival and OS.

Characteristic	Patients at Risk	DFS				OS			
		Univariable Analysis		Multivariable Analysis		Univariable Analysis		Multivariable Analysis	
		HR (95% CI)	*p*-Value	HR (95% CI)	*p*-Value	HR (95% CI)	*p*-Value	HR (95% CI)	*p*-Value
Surgical approach Laparoscopic Robotics	275 146	1 [Reference] 0.57 (0.21–1.56)	0.275	1 [Reference] 1.19 (0.39–3.65)	0.765	1 [Reference] 0.58 (0.12–2.81)	0.501	1 [Reference] 1.35 (0.22–8.5)	0.746
Age at diagnosis <65 ≥65	357 64	1 [Reference] 2.48 (1.01–6.08)	0.048	1 [Reference] 1.23 (0.37–4.07)	0.733	1 [Reference] 0.64 (0.08–5.09)	0.670	NE	NE
BMI <30 ≥30	349 72	1 [Reference] 1.12 (0.41–3.03)	0.829	1 [Reference] 1.54 (0.48–4.88)	0.466	1 [Reference] 0.45 (0.06–3.62)	0.455	1 [Reference] 1.23 (0.12–12.71)	0.864
Stage Stage Ia Stage Ib Stage II Stage III	295 63 25 29	1 [Reference] 1.19 (0.39–3.57) 0.68 (0.09–5.13) 1.08 (0.14–8.22)	0.763 0.706 0.938	1 [Reference] 0.72 (0.11–4.72) 0.61 (0.05–7.81) NE	0.734 0.706 NE	1 [Reference] 0.82 (0.1–6.98) 1.82 (0.21–15.61) 7.32 (1.41–37.93)	0.852 0.584 0.018	1 [Reference] 0.74 (0.03–16.88) NE 1.27 (0.04–38.75)	0.853 NE 0.89
Grade Grade 1 Grade 2 Garde 3	214 117 57	1 [Reference] 2.20 (0.74–6.56) 3.62 (1.1–11.9)	0.155 0.034	1 [Reference] 2.82 (0.91–8.75) 2.04 (0.27–15.2)	0.073 0.488	1 [Reference] 1.87 (0.26–13.28) 6.57 (1.1–39.37)	0.531 0.039	1 [Reference] 1.53 (0.16–15.16) 1.98 (0.09–44.14)	0.714 0.666
Histopathology Endometrioid Non-endometrioid	367 54	1 [Reference] 4.63 (1.94–11.07)	<0.001	1 [Reference] 4.97 (0.7–35.38)	0.11	1 [Reference] 6.22 (1.67–23.25)	0.006	1 [Reference] 2.22 (0.07–67.65)	0.647
Lympho-vascular space invasion Negative Positive	325 87	1 [Reference] 3.02 (1.29–7.06)	0.011	1 [Reference] 5.86 (1.79–19.17)	0.003	1 [Reference] 2.38 (0.57–9.95)	0.011	1 [Reference] 5.86 (1.79–19.17)	0.852
Adjuvant treatment Follow-up Radiotherapy Chemotherapy CCRT	281 28 38 74	1 [Reference] 0.65 (0.08–4.91) 1.58 (0.36–6.98) 1.27 (0.46–3.52)	0.672 0.549 0.650	1 [Reference] NE 0.2 (0.01–3.05) 0.47 (0.07–3.19)	NE 0.247 0.437	1 [Reference] NE 10.15 (2.21–6.74) 1.59 (0.29–8.7)	0.003 0.592	1 [Reference] 9.06 (0.49–168.6) 1.93 (0.09–39.2)	NE 0.140 0.670

## Data Availability

The data for this study are available from the corresponding author on request.
